# Flow goes forward and cells step backward: endothelial migration

**DOI:** 10.1038/s12276-022-00785-1

**Published:** 2022-06-14

**Authors:** Heon-Woo Lee, Jae Hun Shin, Michael Simons

**Affiliations:** 1 Yale Cardiovascular Research Center, Section of Cardiovascular Medicine, Department of Internal Medicine, Yale University School of Medicine, New Haven, CT USA; 2 Department of Immunobiology, Yale University School of Medicine, New Haven, CT USA; 3 Department of Cell Biology, Yale University School of Medicine, New Haven, CT 06520 USA

**Keywords:** Cell lineage, Chemotaxis

## Abstract

Systemic and pulmonary circulations constitute a complex organ that serves multiple important biological functions. Consequently, any pathological processing affecting the vasculature can have profound systemic ramifications. Endothelial and smooth muscle are the two principal cell types composing blood vessels. Critically, endothelial proliferation and migration are central to the formation and expansion of the vasculature both during embryonic development and in adult tissues. Endothelial populations are quite heterogeneous and are both vasculature type- and organ-specific. There are profound molecular, functional, and phenotypic differences between arterial, venular and capillary endothelial cells and endothelial cells in different organs. Given this endothelial cell population diversity, it has been challenging to determine the origin of endothelial cells responsible for the angiogenic expansion of the vasculature. Recent technical advances, such as precise cell fate mapping, time-lapse imaging, genome editing, and single-cell RNA sequencing, have shed new light on the role of venous endothelial cells in angiogenesis under both normal and pathological conditions. Emerging data indicate that venous endothelial cells are unique in their ability to serve as the primary source of endothelial cellular mass during both developmental and pathological angiogenesis. Here, we review recent studies that have improved our understanding of angiogenesis and suggest an updated model of this process.

## Blood vessel formation: Migratory cells lining veins key to growth

Cells that line the inside of veins possess a unique ability to grow new blood vessels and a better understanding of these cells could lead to new treatments for cancer, autoimmunity and other diseases associated with abnormal blood vessel formation. Michael Simons and colleagues from Yale University School of Medicine in New Haven, USA, review the attributes of venous endothelial cells, such as their unique ability to proliferate and migrate against blood flow, and then to form new intricate networks of minute blood vessels, in response to appropriate signals. The authors discuss emerging evidence implicating these cells in a variety of diseases, and suggest that drugs aimed at modulating the molecular function or migratory activities of venous endothelial cells could be used to correct abnormal blood vessel expansion.

## Introduction

The term angiogenesis refers to the process of expansion of a preexisting vascular network. It requires the precise control and coordination of the proliferation, migration, and differentiation of endothelial cells (ECs)^1^. Endothelial cells can be categorized into several subtypes depending on the type of blood vessels they are found in and on their anatomic location. The three principal types of blood endothelial cells are arterial, venous, and capillary cells, and lymphatic endothelial cells form a distinctly different endothelial subpopulation^2–4^. In addition to these well-defined endothelial populations, there is also a population of endothelial tip cells. These cells are characterized by both their specific location at the front of the forming vascular network and the expression of specific genetic programs. Unlike arterial, venous, and capillary endothelial populations, the tip cell population is transient, and any endothelial cells at the vascular edge may assume tip cell features^5–7^.

Because these different endothelial subtypes are exposed to distinct local microenvironments, including factors such as oxygen level, nutrients, and mechanical forces, from the flow of blood, it is not surprising that each endothelial subtype has a distinct gene expression pattern. Other factors, such as functional specialization (e.g., filtration vs. barrier function) or anatomic location (e.g., liver vs. brain), also have a distinct genetic imprint. Given this endothelial diversity, the source of endothelial cells responsible for angiogenic vasculature expansion remains poorly defined.

A recent study^8^ reported that venous endothelial cells are the primary source of endothelial cells responsible for angiogenic expansion both during embryonic development and in disease settings. Remarkably, venous endothelial cells are the only type of endothelial subtype capable of responding to hypoxia-induced angiogenic signaling by proliferating and migrating against the blood flow to form the new vascular bed. Abnormalities in this process account for pathological abnormalities, such as arteriovenous malformation (AVM) or excessive angiogenesis, that are seen in diabetic retinopathy.

Several recent observations also suggest that the role of venous endothelial cells is conserved throughout different settings and species. Given that venous endothelial cells are the primary cellular source of angiogenesis, a better understanding of their biological features will help to facilitate the development of pro- and antiangiogenic therapies. In this review, we will provide a summary of recent advances in this field that focus on endothelial migration and the primary angiogenic role played by the venous endothelium.

## Traditional angiogenesis model

**Fig. 1:**
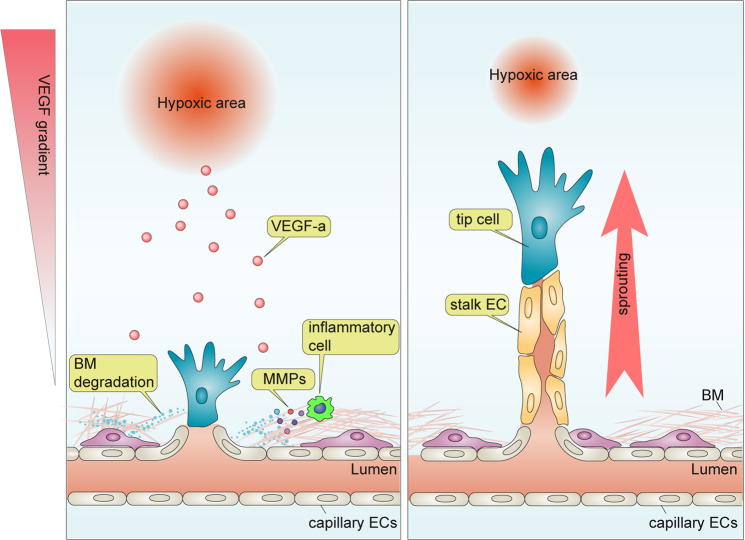
Traditional model for angiogenesis. Cells under hypoxia increase the expression of vascular endothelial growth factor (VEGF-A). This induces vascular stalk formation from the responding capillary bed and stimulates the recruitment of proinflammatory cells, including circulating and tissue-residing macrophages and T cells. The latter is largely responsible for the local production of MMPs that break the extracellular matrix and facilitate endothelial cell migration. Depending on their position in the angiogenic sprout, endothelial cells differentiate into tip or stalk cells. Tip cells at the vascular front sense the VEGF gradient and direct the growing sprout toward its source. Stalk cells follow behind the tip and form an interconnected vascular lumen.

Starting with the work of J Folkman, the concept of angiogenesis as a response to hypoxia has taken hold in this field^9–13^. Thus, the hypoxic environment in a growing tumor or in the developing retina is seen as a source of the principle angiogenesis driver vascular endothelial growth factor A (VEGF-A). It is produced locally due to increased expression of hypoxia-inducible factor 1 alpha (HIF1α), which is a transcription factor regulating VEGF-A expression. This increased VEGF-A production stimulates the growth of new vasculature that, once formed, increases oxygen supply, relieves local ischemia, and arrests further vessel growth (Fig. 1). One important feature of this model is the central role played by a VEGF concentration gradient between the area of maximal VEGF production in the ischemic zone at one end and the “responding” capillary bed at the other (Fig. 1). These capillary endothelial cells respond by sprouting and migrating toward the gradient. The leading cells in the sprout acquire tip cell identity, and the supporting cells acquire a “stalk cell” phenotype. These extending vascular tip/stalk structures eventually form a new capillary bed, thereby restoring blood flow to the formerly ischemic zone (Fig. 1).

In this model, tip cells, a specialized but transient form of endothelial cells characterized by the expression of high levels of VEGF receptor 2 (VEGFR2) and the absence of a lumen, play a central role in the angiogenic response. VEGF binding to VEGFR2 on tip cells results in the activation of a number of tip endothelial cell-specific genes^14,15^. These include growth factors (PDGFB^16^, ANG2^17^, and APLN^18^), angiogenic receptors (PDGFRβ^17^, VEGFR1^19^, VEGFR3^20^, UNC5B^19^, and CXCR4^18^), and others (DLL4^21,22^ and ESM1^18^).

This distinct gene expression profile enables tip endothelial cells to sense angiogenic cues and the VEGF gradient and guide newly formed angiogenic sprouts toward the hypoxic area^23^. While tip endothelial cells guide the growing sprout, endothelial cells located just behind the tip cell position, known as stalk cells, form the body of the angiogenic sprout containing the blood vessel lumen. Similar to tip cells, stalk cells are also a transient endothelial cell subtype. In contrast to tip cells, stalk cells have fewer filopodia and reduced levels of VEGFR2. They support the extension of angiogenic sprouts, forming the lumen of the new vessel and maintaining a connection with the preexisting capillary bed^24–27^. Importantly, fate-mapping studies confirmed the contribution of tip cells to angiogenesis, demonstrating that a high proportion of the newly formed arterial endothelium is of tip cell origin^28,29^.

To prevent a runaway train of angiogenesis, the process of tip vs. stalk cell specification and angiogenic sprouting is regulated by the activation of Notch signaling at endothelial cell–cell contact sites. An endothelial cell exposed to VEGF upregulates its expression of the Notch ligand Delta-like 4 (DLL4), thereby acquiring a tip cell fate. This newly minted tip cell interacts with NOTCH receptors on adjacent endothelial cells, resulting in NOTCH cleavage and the release of the intracellular Notch intracellular domain (NICD). NICD translocates into the nucleus, binds to RBPJ, and then activates NOTCH target genes. This converts endothelial cells with activated Notch signaling into stalk cells, limiting the number of newly formed sprouts. This promotes vascular quiescence, stabilizes EC-EC junctions, and suppresses proliferation^24,30,31^. Overall, endothelial NOTCH activation prevents excessive angiogenesis by reducing the overall number of tip endothelial cells. In the absence of this mechanism, such as in DLL4+/− mice^23^, an excessive number of new vessels are formed, resulting in defective tissue perfusion.

One shortcoming of the traditional tip/stalk specification-centered concept of angiogenesis discussed above (Fig. 1) is that it is fairly static and does not take into account ongoing endothelial cell proliferation and migration. Since any angiogenic expansion requires an adequate supply of endothelial cells to support growth of the new vasculature, the preexisting endothelium must proliferate and migrate toward the site of angiogenesis. This new wave of endothelial cells provides a source of tip and stalk cells that guide the growth of new vasculature. With this in mind, one key unresolved gap in our understanding of angiogenesis is the source of endothelial cells that is driving it.

An important step in any angiogenic response is the initiation of endothelial migration. This requires the degradation of the existing extracellular matrix and the basement membrane surrounding quiescent endothelial cells, thereby allowing them to move^32,33^. While there are a number of enzymes capable of digesting different extracellular matrix components, matrix metalloproteinases (MMPs) are particularly important. Endothelial activation in response to VEGF and the influx and activation of proinflammatory cells, such as circulating and tissue-residing macrophages and T cells, leads to the local production of MMPs (Fig. 1). This enables the initiation of endothelium migration in response to the appropriate signals^32,34,35^. Because endothelial cells in the capillary bed are embedded in thinner basement membranes than those in arteries and veins^36,37^, it has been hypothesized that capillary endothelial cells are the source of endothelial expansion. However, this idea has been challenged by recent findings that venous endothelial cells are the primary source of endothelial cells for angiogenic expansion during developmental and pathological angiogenesis^8^.

## Regulation of the angiogenic response by flow

**Fig. 2:**
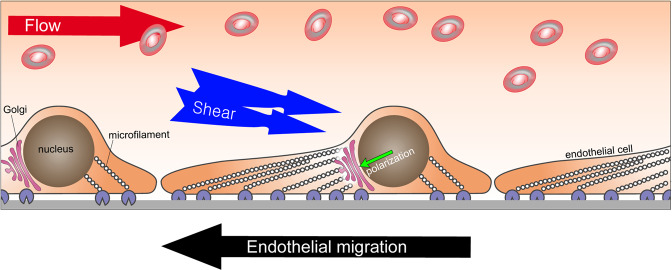
Endothelial migration under blood flow. Flow-induced shear stress provides a directional cue for endothelial migration that occurs against the flow of blood. Endothelial cells under laminar flow display aligned microfilament networks and upstream polarization of the Golgi apparatus (GA).

In addition to sensing VEGF gradients and responding to other circulating or locally present growth factors, endothelial cells are also exposed to dynamic forces generated by the flow of blood. The ability to sense and respond to these forces is a key feature of the endothelium and governs much of its behavior (Fig. 2)^38,39^. Laminar blood flow induces the endothelial cell synthesis of nitric oxide (NO), the extent of which is positively correlated with the flow intensity^40^. Thus, NO induces the dilation of blood vessels, thereby lowering vascular resistance and facilitating tissue perfusion. In addition, flow-mediated shear stress regulates the expression of arterial and venous marker genes, including DLL4^41^, EphrinB2^42^, and KLF4^43^, inflammatory markers (KLF2^44^, ICAM1^45^, and E-selectin^45^), and vasodilation factors (COX2 and PGI2^46^), among others. Endothelial cells under laminar flow are polarized and undergo extensive rearrangements of intracellular organelles^47^. One such key cytoskeletal rearrangement is the polarization of the Golgi apparatus^48^. This is important in the context of cell migration. The orientation of the Golgi complex is coupled to the direction of cell migration and can be used as an indicator of where the cell in question is going. This has been described in a number of cell types, including fibroblasts^49^, natural killer (NK) cells^50^, epithelial cells^51^, and cancer cells^52^. For example, a study of Golgi complex polarization in fibroblasts during “wound”-induced migration in vitro showed that cells located far from the wound exhibited random Golgi orientation. However, the Golgi in cells near the wound’s edge were polarized toward the wound, indicating the direction of their migration^53^.

Likewise, endothelial cells under laminar flow display an aligned microfilament network and an upstream polarization of the Golgi (Fig. 2). The Golgi complex in bovine carotid endothelial cells showed upstream polarization when subjected to laminar flow, while those under confluent and static conditions showed an unpolarized orientation^54^.

The upstream polarization of the Golgi towards the flow direction was also observed in human umbilical vein endothelial cells^55–57^, coronary artery endothelial cells^58^, and the aortic endothelium^59^. These findings suggest that it is a conserved feature among endothelial cells from different vascular beds. A study of endothelial Golgi-nucleus polarization under different magnitudes of fluid flow showed that in the majority of cells, the Golgi complex was polarized regardless of the flow magnitude.

In addition to these in vitro studies, recent studies using zebrafish clearly showed upstream polarization of endothelial cells in vivo. Using transgenic zebrafish expressing endothelial-specific nEGFP (nuclear localizing EGFP; Tg(kdrl:NLS-EGFP)) and endothelial-specific mCherry-labeled Golgi marker (Tg(fli1a:B4GALT1-mCherry), Kwon et al.^60^ demonstrated the upstream polarization of endothelial cells in the dorsal aorta and arterial/venous intersegmental vessels. Endothelial cells in blood vessels with flow rapidly polarized once the flow started, while those without flow showed random polarization. These findings indicate that blood flow is the paramount factor determining the direction of endothelial polarization in vivo. In line with this observation, the upstream polarization of endothelial cells has been reported in a number of in vivo models and settings. In the postnatal retina vasculature, Golgi marker (Golph4^61^ and GM130^8,48^) immunostaining showed upstream polarization in both arteries and veins. Endothelial cells in various mouse organs, including carotid arteries, jugular veins, thoracic aorta and the vena cava^48,57^, femoral^62^, and coronary arteries^63^, are also polarized against blood flow. Other species, including pigs (thoracic aorta and inferior vena cava)^64^ and rabbits (inferior vena cava)^64^, have also been reported to display the upstream polarization of endothelial cells against flow.

**Fig. 3:**
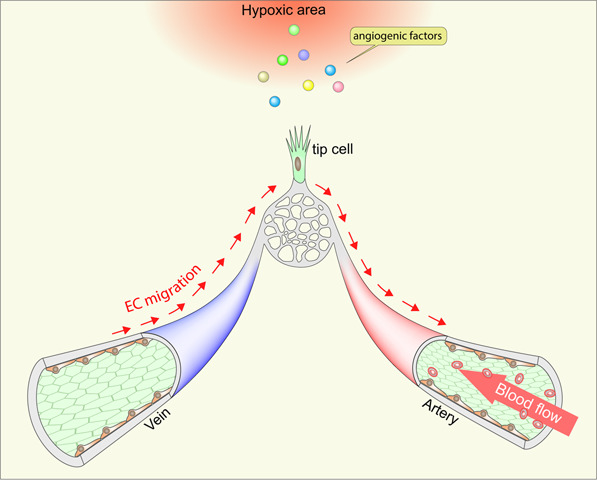
Endothelial migration during angiogenesis. Venous endothelial cells migrate against blood flow and differentiate into tip and arterial endothelial cells. Cells in hypoxic areas release angiogenic factors, including VEGF-A, to initiate angiogenesis. This initiates venous endothelium migration into the vascular front via reverse migration. Venous endothelium-derived tip cells continue reverse migration and differentiate into arterial endothelial cells. Venous endothelial cells act as a primary source of ECs for angiogenic expansion through reverse migration.

Upstream polarization implies a reverse migration of endothelial cells against the bloodstream. The first in vivo evidence supporting reverse migration was real-time imaging of the mouse yolk sac from transgenic mice carrying fluorescence-labeled endothelial cell membranes (Flk1-myr::mCherry) and nuclei (Flk1-H2B::eYFP)^65^. Using live confocal imaging of a transgenic mouse embryo, Udan et al.^65^ demonstrated that endothelial cells in capillaries migrate against the bloodstream. They end up in large arteries and contribute to the expansion of the artery diameter. Moreover, reduced blood flow^66,67^ in *Mlc2a*^−/−^ (myosin regulatory light chain 2) mice showed that the hemodynamic force is essential for the reverse migration of endothelial cells and their recruitment into arteries^65^. Reverse migration has also been reported using a fin regeneration model in a transgenic zebrafish line carrying reporter genes for endothelial nuclei (*Tg(fli1a:nEGFP)*_*y7*_) and arterial-EC membranes (*Tg(–0.8flt1:RFP)*_*hu5333*_)^68^. Time-lapse confocal imaging over 24 h demonstrated that venous endothelial cells migrated against the bloodstream, took over tip cell positions, and then continued the reverse migration toward the arterial bed. Likewise, endothelial cells in mammals also showed similar migration patterns. Time-course lineage tracing with tip cell-specific inducible Cre recombinase-expressing mice (*Esm1(BAC)CreER*^*T2*^*; R26*^*mTmG*^) demonstrated that tip cells in postnatal retina vasculature are recruited into arteries but not veins, supporting the reverse migration paradigm^28,68^. The most recent study reported that the reverse migration of the endothelium is a conserved feature among different organs, including the brain, retina and dermis, using a venous endothelium-specific inducible Cre line (*Gm5127(BAC)CreER*^*T2*^*;R26*^*mTmG*^)^8^. Lee et al.^8^ found a novel venous endothelium marker and generated venous EC-specific inducible Cre and Dre driver mouse lines. Time-course lineage tracing revealed that venous endothelial cells migrate against the bloodstream and sequentially differentiate into capillary, tip, and arterial endothelial cells^8^. Strikingly, the majority of endothelial cells (~80%) in the postnatal angiogenic stage originate from the venous endothelium, indicating that venous endothelial cells are the primary source of EC during angiogenic expansion (Fig. 3).

Venous endothelial cells are more mitotically active than those in other vascular beds^69^. This implies that highly proliferating venous endothelial cells migrate against the bloodstream, differentiate into other endothelial subtypes, and serve as the primary source of endothelial cells for the expansion of the vascular network (Fig. 3). Venous endothelial cells in the adult mouse vasculature are quiescent, do not migrate and lose their mitotic activity. Thus, pathological angiogenic processes in the adult stage must require a stimulus, such as an active angiogenic process, to restore their proliferative/migratory abilities. Indeed, this has been observed in zebrafish. Zebrafish reach sexual maturity by 3 months of age^70^, and as in adult mice, the endothelial mitotic activity in adult zebrafish is significantly decreased^71^. Induction of a fin injury (fin regeneration model) induces the reverse migration of venous endothelial cells with mitotic activity in adult zebrafish^68^. This demonstrates that quiescent venous endothelial cells can reinitiate proliferation and reverse migration in response to pathological stimuli.

## Flow-independent migration of endothelial cells

**Fig. 4:**
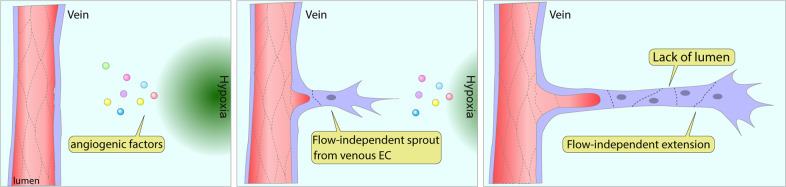
Flow-independent sprouting angiogenesis originates from venous endothelial cells. Among the three different endothelial subtypes, arterial, capillary and venous, only venous endothelial cells respond to angiogenic factors released from the hypoxic area. Initial sprouts from preexisting veins do not have a lumen for blood circulation, and the process of lumenization follows vascular stalk formation.

**Fig. 5:**
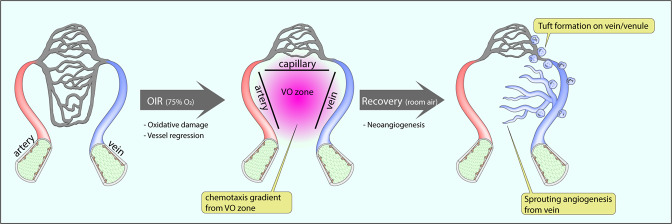
Venous endothelial cells and angiogenesis in oxygen-induced retinopathy (OIR). A transient high-oxygen environment (75% oxygen) induces oxidation damage to preexisting vessels. This leads to vascular regression and the formation of the avascular vaso-obliteration (VO) zone, which is surrounded by three different endothelial subtypes (arterial, venous, and capillary). A return to the ambient O_2_ concentration initiates angiogenic sprouting to revascularize the VO zone, a process that involves sprouting angiogenesis from venous ECs and neovascular tuft formation on veins/venules.

While hemodynamic forces certainly play an important role in activating and directing endothelium migration, other factors are also involved. In particular, angiogenic factors, such as VEGF released by ischemic tissues, are also capable of stimulating endothelial migration (Fig. 4). In this process, the endothelial cells closest to the chemotaxis gradient are selected as tip cells and initiate angiogenic sprouting. Although tip cells in the vascular front do not have direct contact with flow due to the lack of lumen^72^ (Fig. 4), tip cells actively guide angiogenic sprouting by sensing angiogenic cues from the hypoxic area^16^. This process is subjected to a chemotaxis-dependent process rather than flow-directed migration^73^. Since capillary vessels are generally located at the periphery of the vascular network, endothelial cells in those vascular beds have been considered the endothelial subtype closest to the chemotaxis gradient. If that is the case, these capillary endothelial cells would be the major endothelial subtype responding to the VEGF gradient. However, our recent study^8^ showed that only venous endothelial cells, but not other subtypes, respond to a hypoxia-induced chemotaxis gradient (Fig. 4). This finding is similar to the findings demonstrated during retinal vasculature development and in an OIR model. The OIR is particularly illustrative here. In this model, an ischemic vaso-obliteration zone, induced by exposure to a high O2 environment, is surrounded by three different vascular beds (capillary, artery, and vein). Immunocytochemistry shows that the initiation of angiogenic sprouting only occurs from the venous bed, indicating that capillary and arterial endothelial cells do not respond to the chemotaxis gradient. Furthermore, cell fate tracing confirms that ~80% of tip cells in the VO zone are of venous endothelium origin. This finding demonstrates that the venous endothelium is the primary subtype responsible for tip cell differentiation and endothelial expansion during the OIR-induced neoangiogenic process (Fig. 5).

**Fig. 6:**
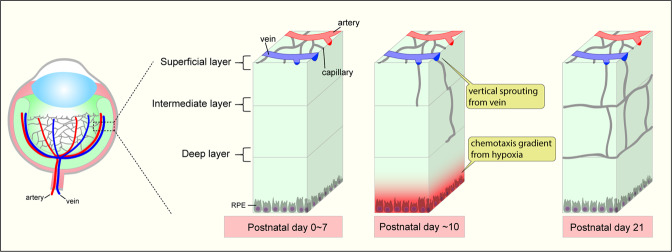
Venous endothelial cells and vertical sprouting in retina vessel development. After P8 (postnatal day 8), the expanding vascular network on the superficial layer of the retina reaches its periphery and then begins to dive to form intermediate/deep retinal vessel layers. Chemotaxis from retinal pigment epithelial cells (RPE) and cells under deep layers exposed to a hypoxia-generated chemotaxis gradient drives vertical sprouting from the vessels in the superficial layer. Venous endothelial cells are the primary subtype of cells that initiate vertical angiogenic sprouting.

Similar observations apply to the role of the venous endothelium during the formation of the retinal vasculature (Fig. 6). After birth, vasculature in the retinal superficial layer starts angiogenic expansion from the optic disk to the retinal periphery. Around postnatal day 8 (P8), the superficial vasculature reaches the periphery of the retina. There, it changes the direction of expansion and initiates vertical sprouting that “dives” into deep and intermediate layers (Fig. 6). RPE cells under the outer nuclear layer are exposed to hypoxia due to the lack of oxygen and generate a chemotactic gradient to attract endothelial cells from the superficial layer. Notably, initial neoangiogenic vessels in the deep layer at P10 are predominantly found beneath veins, implying that the venous endothelium once again plays a major role in the angiogenic expansion. Indeed, analysis of venous endothelium-derived ECs using venous EC tracing in mice showed that ~80% of endothelial cells in the deep layer were derived from venous endothelium^8^. Given that vertical sprouting is initiated by a chemotaxis gradient rather than flow-directed migration, the distinct features of venous endothelial cells responding to chemotaxis might be shared between OIR-induced pathological angiogenesis and developmental processes.

## Venous endothelial cells and pathological angiogenesis

In addition to serving a useful physiological role by restoring tissue perfusion, angiogenesis also plays a key role in the progression of various diseases, including cancer and various retinopathies. However, the source of neo-angiogenic endothelial cells in pathological lesions is poorly understood due to technical limitations. Recent studies have shown that the venous endothelium is the key source of neo-angiogenic endothelial cells during pathological angiogenesis.

### Vascular malformations

**Fig. 7:**
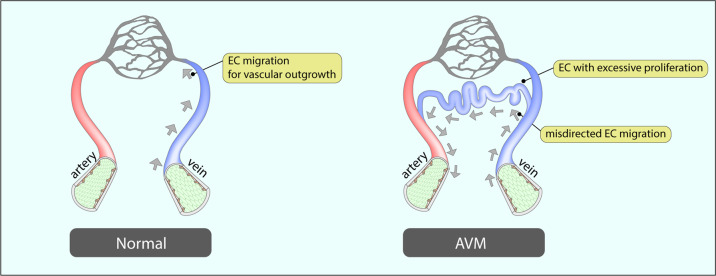
Venous endothelial cells and arteriovenous malformations (AVMs). In a normal vasculature, venous endothelial cells migrate against blood flow toward the angiogenic front for vascular outgrowth. In AVMs, the migration of venous endothelial cells is disturbed, and the speed of migration is accelerated compared to normal venous endothelial cells. Hyperproliferation of misdirected venous endothelial cells results in abnormal connections between arteries and veins.

Arteriovenous malformations (AVMs) are one of the most widely used models to investigate mechanistic details of endothelial dysfunction in angiogenesis because the mouse AVM model provides a highly detailed view of the AVM structure in the superficial layer of the retinal vasculature. Arteriovenous malformations are a common vascular disorder characterized by an abnormal connection between an artery and a vein (AV shunt). They are caused by loss-of-function mutations of genes in the TGFβ/BMP signaling pathway, including ALK1^74^, ENG^75^, and SMAD4^76,77^. The key feature of AVM development is a misdirected endothelial alignment. In particular, the loss of endoglin (*ENG)*, a coreceptor of TGFβ/BMP signaling, alters endothelial shapes under blood flow^78^, and vessels with *ENG* loss developed AV shunts in both zebrafish^78^ and mice^75^. Activin receptor-like kinase 1 (Alk1)-deficient endothelial cells also display polarization defects under flow^56^ that also play a role in the development of AVM^74,79^. SMAD4, a downstream transcription factor of the TGFβ/BMP signaling pathway, is yet another gene involved in the control of endothelial alignment. While normal endothelial cells are polarized and aligned along the axis of the flow, SMAD4-deficient endothelial cells exhibit a lack of alignment and reduced polarization^58^. This observation has also been confirmed in the retinal vasculature in in vivo settings. Misdirected endothelial alignment and polarization result in AVM development in endothelium-specific SMAD4 KO mice (*SMAD4*^*iECKO*^*)*^8,76,77^. While the venous endothelial Golgi complex is polarized toward the retinal periphery in normal mice, those in *SMAD4*^*iECKO*^ mice polarized toward arteries displaying venous endothelial cells. Thus, in *SMAD4*^*iECKO*^ mice, the polarization is abnormally directed (Fig. 7)^8^.

Growing evidence indicates that venous endothelial cells also play a crucial role in the development of AVMs. For instance, endothelial cells in AV shunt lesions are known to have a venous identity. Venous endothelium marker (EphrinB2)^75^ and venous-capillary marker (Endomucin)^80^ but not arterial markers (Jag1 and Aplnr) are highly expressed in AV shunt lesions of *ENG*^*iECKO*^ mice. AVMs in *SMAD4*^*iECKO*^ and *ALK1*^*iECKO*^ mice also show upregulated expression of venous endothelial markers, including EphrinB2 and Nrp2^74,76,77^. Interestingly, deletion of *ENG* with venous/capillary endothelium-specific Cre (*Apj-CreER*^*T2*^) and pan endothelium-specific Cre (*Cdh5-CreER*^*T2*^) led to a similar incidence and frequency of AVMs, indicating that arterial *ENG* does not protect AVM development. Likewise, deletion of ALK1 with venous/capillary endothelium-specific Cre (*Mfsd2a-CreER*^*T2*^) leads to AVM development but not tip- (*Esm1-CreER*^*T2*^) or arterial EC (*Bmx-CreER*^*T2*^)-specific deletion^56^. These in vivo findings imply that endothelial cells in capillaries and/or veins are the main drivers of the development of AVMs.

Using fate mapping that employed a dual Cre and Dre recombinase system that deletes SMAD4 in the pan endothelium and simultaneously labels venous endothelial cells by tamoxifen administration (*SMAD4*^*fx/fx*^*;Cdh5-CreER*^*T2*^*;Gm5127-DreERT2; Rosa26*^*RC::RG*^), Lee et al.^8^ traced the origin of endothelial cells in AVM lesions to the venous endothelium. Time-course analysis of AVMs showed that AVM formation is initiated by abnormal sprouting from veins and that the majority of endothelial cells in AVMs originate from venous endothelial cells^8^. The lack of polarization/alignment in SMAD4-deficient endothelial cells against flow^58^ and the excessive proliferation of endothelial cells in AVM lesions^8^ suggest that AVMs are consequences of the undirected migration of excessively proliferating venous endothelial cells. Recent single-cell RNA sequencing analysis of endothelial cells in a cerebral cavernous malformation (CCM) model also revealed that those in CCM lesions originate from a subset of venous/capillary endothelial cells^81^, indicating that the role of venous endothelial cells in malformation is not limited to AVMs. Jin et al.^82^ reported that the mosaic loss of ENG (by low-dose tamoxifen injection in *ENG*^*iECKO*^ mice) resulted in initial AVM formation in arterioles. Identification of the origin of ECs in those lesions using EC subtype-specific reporter lines may explain the discrepancy in the ENG-deficient setting.

### Oxygen-induced retinopathy

The oxygen-induced retinopathy (OIR) model has been widely used in cardiovascular research. While it closely resembles retinopathy of prematurity^83,84^, its vascular pathologies also showed relevance to other ischemic diseases, including proliferative diabetic retinopathy^85^ and retinal vein occlusion^86^. Exposing neonatal pups to hyperoxic (75% oxygen) conditions for several days leads to the formation of a vaso-obliteration (VO) zone, and returning them to the room air triggers neovascularization to alleviate the VO zone (Fig. 5)^8^. As discussed above, venous endothelial cells are the exclusive source of ECs in this neovascularization process (Fig. 5). Vascular tufts are a typical feature of the OIR. During the neoangiogenic repair process in the OIR model, a retinal vasculature forms neovascular tufts at the border between the VO zone and the vascularized area, specifically around veins and venules (Fig. 5)^87^. The tuft formation process is known as pathological angiogenesis and has made the OIR model a key tool for the study of vascular pathology in ischemic retinopathies^88^. Interestingly, genetic tracing of venous endothelium has revealed that those cells are the primary endothelial source for the formation of vascular tufts in the OIR model^8^.

### Wound healing

**Fig. 8:**
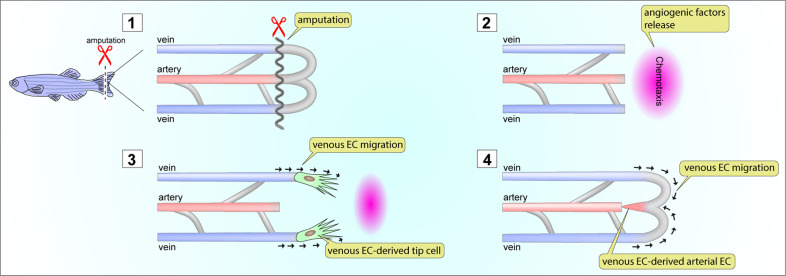
Venous endothelial cells and wound healing. Zebrafish regenerate amputated caudal fins. During the regeneration process, reactivation of endothelial migration is essential to support angiogenic activity. After amputation (1), chemotaxis from the wounded area attracts ECs (2). Among the three different EC subtypes, only venous ECs respond to angiogenic factors from the wounded area and initiate migration into the wound (3). During the migration of venous ECs, they differentiate into tip ECs (3). Then, venous EC-derived tip ECs change the direction of migration, migrate toward the artery and differentiate into arterial ECs (4).

Angiogenesis plays an important role in wound healing. Newly formed blood vessels invade the wound and facilitate its resorption and closure^89,90^. Zebrafish have been extensively utilized as an in vivo model of angiogenesis during wound healing for over a decade because of their ability to regenerate caudal fins after amputation^91^. Major players involved in the angiogenic process (i.e., growth factors and their receptors) are conserved between species and function similarly^92,93^. The availability of various genetically modified zebrafish lines expressing fluorescent proteins in each endothelial subtype (venous-^94^, arterial-^95,96^, and pan endothelium^97^) made this model more attractive for the intravital imaging of postinjury angiogenesis^98,99^. Amputation of the caudal fin activates endothelial cells in preexisting vascular beds to invade the wound area and establish a new vascular network (Fig. 8). Time-lapse image analysis of zebrafish fin regeneration using genetic lineage tracing strategies has identified venous endothelial cells as the primary endothelial subtypes invading the wounded area^68,100^. During the regeneration process, venous endothelial cells migrate toward the wounded area. There, they differentiate into tip cells and eventually differentiate into arterial endothelial cells (Fig. 8)^68^. Given that the venous endothelium is the primary endothelial source in angiogenesis in mice, these regeneration models imply that the role of venous endothelial cells during angiogenic expansion is also conserved in different species.

w are still largely unexplored. Recent advances in scRNA-sequencing technology have enabled large-scale sequencing and analysis of various endothelial cell populations in cancer. Interestingly, studies of the tumor endothelium have revealed that the numbers of activated postcapillary venous endothelial cells were increased in the lung tissues of cancer patients compared to healthy controls^101^. A venous marker (NR2F2, a.k.a. COUP-TFII) is also highly expressed in the tumor endothelium, implying the importance of the venous endothelium in tumor angiogenesis^101^.

## Future perspective and conclusion

Despite three decades of development, the success of pro- and anti-angiogenic therapies has been rather modest. The best results achieved in retinal diseases were from the use of anti-VEGF therapies that resulted in the significant preservation and improvement of vision^102,103^. Clearly, alternative strategies are needed to overcome the current limitations. To date, most efforts to control the process of angiogenic vascular growth have mainly focused on VEGF-A and its receptors, while other factors regulating this process have been largely ignored. Recently, a growing body of evidence has started indicating the importance of venous endothelial cells in developmental and pathological angiogenesis in a number of pathological settings. Venous endothelial cells are distinct from other endothelial subtypes in that they possess high mitotic activity, are able to migrate against blood flow, and can assume the molecular and functional identity of other endothelial subtypes. Despite much progress in understanding the role of the venous endothelium in various developmental and pathological settings, elucidation of the molecular mechanisms that confer these features on the venous endothelium has only just begun. One important feature of this endothelial subtype is that quiescent venous endothelium, unlike other endothelial subtypes, can be reprogrammed to activate their ability to migrate in response to angiogenic and mechanical stimuli. This implies the presence of specific molecular control mechanisms that regulate the reactivation process and migratory activity in the venous endothelium. A better understanding of these mechanisms and why they are unique to the venous endothelium may enable us to develop better therapeutic strategies for angiogenesis-related diseases.
